# Expected clinical and patient-centered outcomes associated with different rehabilitation strategies for severe maxillary atrophy: an international cross-sectional survey

**DOI:** 10.3389/froh.2026.1873284

**Published:** 2026-09-16

**Authors:** Giulio Gasparini, Mattia Todaro, Edoardo Papa, Marco Gaetano Amato, Paolo De Angelis, Alessandro Moro, Carlo Lajolo, Gianluca Nicolai, Edoardo Rella, Roberto Boniello, Xavier Moix Gil, Edoardo Staderini, Sofia Gasparini, Federico Perquoti, Gianmarco Spinozzi, Veronica Santoro, Daniele Di Carlo, Giuliano Ascani, Paolo Francesco Manicone, Maurizio Diego Foresti, Maurizio Perugini, Francesca Azzuni, The Collaborative Working Group, Martinna Bertolini, Gianmarco Saponaro

**Affiliations:** 1 Pre-prosthetic Surgery Unit, Fondazione Policlinico Agostino Gemelli IRCCS Hospital, Rome, Italy; 2 Maxillo-Facial Surgery Unit, IRCSS “A. Gemelli” Foundation-Catholic University of the Sacred Heart, Rome, Italy; 3 Department of Emergency and Acceptance, UOSD Maxillofacial Surgery, University of Rome Tor Vergata, Rome, Italy; 4 Department of Head and Neck, Oral Surgery and Implantology Unit, Institute of Clinical Dentistry, Catholic University of the Sacred Heart, Polyclinic Foundation, Rome, Italy; 5 Head and Neck Department, “Fondazione Policlinico Universitario A. Gemelli—IRCCS,” School of Dentistry, Università Cattolica del Sacro Cuore, Rome, Italy; 6 Department of Head and Neck and Sensory Organs, Division of Oral Surgery and Implantology, Fondazione Policlinico Universitario A. Gemelli IRCCS-Università Cattolica del Sacro Cuore, Rome, Italy; 7 IRCCS, Fondazione Policlinico Universitario Agostino Gemelli, Roma, Italy; 8 Department of Orthodontics, Università Cattolica del Sacro Cuore, Rome, Italy; 9 Division of Maxillo-Facial Surgery, Department of Medicine, Surgery and Dentistry, ASST Santi Paolo e Carlo Hospital, University of Milan, Milan, Italy; 10 Department of Maxillofacial Surgery, Spirito Santo Hospital, Pescara, Italy; 11 Division of Prosthodontics—Institute of Clinical Dentistry, Department of Neuroscience, Sensory Organs and Chest, Fondazione Policlinico Universitario A. Gemelli—IRCCS, School of Dentistry, Universita’ Cattolica del Sacro Cuore., Roma, Italy; 12 Maxillo-Facial Surgery Unit, Ospedale SS. Trinità, Cagliari, Italy; 13 Department of Maxillo Facial Surgery, Santa Rosa Hospital Viterbo, Viterbo, Italy

**Keywords:** bone grafting, Cawood and Howell Class VI, clinician preferences, patient-specific implants (PSI), pterygoid implants, severe maxilla atrophy, treatment decision—making, zygomatic implants

## Abstract

**Background:**

Severe maxillary atrophy remains one of the most challenging conditions in implant dentistry and maxillofacial surgery. Multiple rehabilitation strategies are currently available, including regenerative procedures, alternative implant-based approaches, and hybrid concepts. Although clinical outcomes have been widely reported, limited information exists regarding clinician-reported expectations associated with different rehabilitation strategies when evaluating the same clinical scenario. The aim of this study was to investigate clinician-reported expectations associated with different rehabilitation approaches for severe maxillary atrophy.

**Materials and methods:**

An international cross-sectional survey was conducted using a standardized anonymized CBCT case classified as Cawood and Howell Class VI. The questionnaire was distributed through professional and academic networks between July and November 2025. Respondents with experience in at least five complex implant-prosthetic rehabilitations per year were included. After selecting their preferred rehabilitation strategy, participants completed a branching questionnaire exploring clinician-reported expectations regarding safety, predictability, immediate loading, implant survival, treatment duration, smile satisfaction, masticatory function, oral hygiene and maintainability, postoperative morbidity, annual maintenance costs, patient satisfaction, and quality-of-life improvement. Descriptive statistics were performed.

**Results:**

A total of 155 eligible clinicians from 33 countries were included. Alternative implant-based strategies were selected by 83 respondents (53.5%), regenerative approaches by 45 (29.0%), and hybrid approaches by 27 (17.4%). Clinicians selecting alternative implant-based strategies more frequently anticipated immediate loading (81.9%), functional rehabilitation within 72 h (83.1%), and implant survival exceeding 95% at five years (57.8%). Respondents selecting regenerative approaches more commonly expected treatment durations of approximately one year (64.4%) and reported highly favorable expectations regarding smile satisfaction (86.7%), patient satisfaction (82.2%), and quality-of-life improvement (97.8%). Across all therapeutic preference groups, clinician-reported expectations regarding masticatory function, patient satisfaction, and quality-of-life improvement were generally favorable. Most respondents anticipated postoperative discomfort lasting between one and four weeks and treatment abandonment rates below 5%.

**Conclusions:**

Clinicians selecting different rehabilitation strategies for severe maxillary atrophy reported distinct expectations regarding treatment timing, predictability, implant survival, aesthetics, maintenance requirements, morbidity, and patient-centered outcomes. Despite these differences, expectations were generally favorable across all therapeutic preference groups, suggesting that multiple rehabilitation pathways are currently perceived as viable treatment options. The findings should be interpreted as clinician-reported expectations rather than direct measurements of clinical or patient-reported outcomes.

## 1 Introduction

Severe maxillary atrophy remains one of the most challenging conditions in implant dentistry and maxillofacial surgery. Advanced resorption of the maxillary alveolar process may compromise implant placement, prosthetic rehabilitation, facial support, and overall oral function, often requiring complex treatment planning and multidisciplinary management. Over recent decades, several therapeutic strategies have been proposed to address this condition, including regenerative procedures with bone grafting, alternative implant-based approaches relying on remote skeletal anchorage, and hybrid treatment concepts combining reconstructive and graftless principles.

The increasing availability of zygomatic implants, pterygoid implants, transnasal implants, tilted implants, and patient-specific subperiosteal implants has expanded the range of treatment options available for patients with severe maxillary atrophy. At the same time, reconstructive procedures based on autogenous bone grafting and guided bone regeneration continue to represent established therapeutic approaches, particularly when restoration of skeletal volume and soft-tissue support are considered important treatment objectives. Previous studies have demonstrated the long-term clinical validity of both regenerative and graftless approaches, highlighting their respective advantages, limitations, and indications in the rehabilitation of severely atrophic maxillae (1–7).

As the number of available therapeutic alternatives has increased, treatment planning has become progressively more complex. In many clinical situations, multiple rehabilitation strategies may be considered technically feasible, each characterized by different biological principles, treatment duration, surgical complexity, prosthetic implications, expected morbidity, and aesthetic objectives. Consequently, clinician decision-making is increasingly influenced not only by anatomical considerations but also by expectations regarding treatment outcomes and patient-centered factors (8–10). Contemporary treatment planning therefore requires balancing objective clinical conditions with anticipated functional, aesthetic, and quality-of-life benefits associated with different rehabilitation strategies.

Although numerous studies have reported clinical outcomes associated with individual rehabilitation techniques, comparatively little information is available regarding how experienced clinicians perceive and anticipate the potential benefits and limitations of different treatment strategies when confronted with the same clinical scenario. Understanding these expectations may provide insight into contemporary therapeutic decision-making and treatment planning strategies in severe maxillary atrophy.

The aim of the present international cross-sectional survey was therefore to investigate clinician-reported expectations associated with different rehabilitation strategies for severe maxillary atrophy. Using a standardized anonymized CBCT case classified as Cawood and Howell Class VI (11), the survey explored clinician perceptions regarding predictability, immediate loading, expected implant survival, treatment duration, aesthetic outcomes, masticatory function, oral hygiene and maintainability, postoperative discomfort, maintenance burden, patient satisfaction, and quality-of-life improvement. The study was designed to describe clinician-reported expectations and perceptions rather than direct clinical outcomes.

## 2 Materials and methods

### 2.1 Study design and general objectives

The present study was designed as an international cross-sectional survey aimed at investigating clinician-reported expectations associated with different rehabilitation strategies for severe maxillary atrophy classified as Cawood and Howell Class VI.

The study was based on a structured online questionnaire developed to collect clinician-reported information regarding perceived treatment characteristics and expected outcomes associated with different rehabilitation strategies for severe maxillary atrophy.

The primary objective was to describe expectations associated with alternative implant-based, regenerative, and hybrid rehabilitation strategies when applied to the same standardized anonymized CBCT case.

Secondary objectives included the evaluation of clinician-reported perceptions regarding safety, predictability, tolerability, possibility of immediate loading, expected implant survival, treatment duration, aesthetic outcomes, masticatory function, oral hygiene and maintainability, postoperative discomfort, treatment dropout due to morbidity, annual maintenance costs, patient satisfaction, and quality-of-life improvement.

The study was designed to investigate clinician-reported expectations and perceptions rather than direct clinical outcomes or patient-reported outcome measures.

### 2.2 Participants and eligibility criteria

The study population consisted of oral surgeons, maxillofacial surgeons, implantologists, prosthodontists, and clinicians involved in advanced implant-prosthetic rehabilitation.

Participants were recruited through direct invitations, scientific mailing lists, professional societies, and international academic and professional networks.

The initial dataset included 178 submitted questionnaires. The predefined eligibility criterion was self-reported experience in complex implant-prosthetic rehabilitation, defined as the treatment of at least five complex rehabilitations per year.

After screening, 23 questionnaires were excluded because respondents reported fewer than five complex rehabilitations per year. The final analysis therefore included 155 eligible responses.

The final sample was categorized into three therapeutic preference groups according to the rehabilitation strategy selected for the standardized clinical case:

- Alternative implant-based strategies: 83 participants (53.5%)
- Regenerative approaches with bone grafting: 45 participants (29.0%)
- Hybrid/combined strategies: 27 participants (17.4%)

Participation was voluntary, unpaid, and independent from commercial sponsorship.

### 2.3 Questionnaire structure and standardized clinical case

The questionnaire was developed in English using the Google Forms platform to facilitate international dissemination and standardized access.

Participants were presented with a standardized clinical case of severe maxillary atrophy classified as Cawood and Howell Class VI. The case was designed to simulate a complex but realistic implant-prosthetic rehabilitation scenario. Representative images of the anonymized CBCT case are provided in Figure 1.

**Figure 1:**
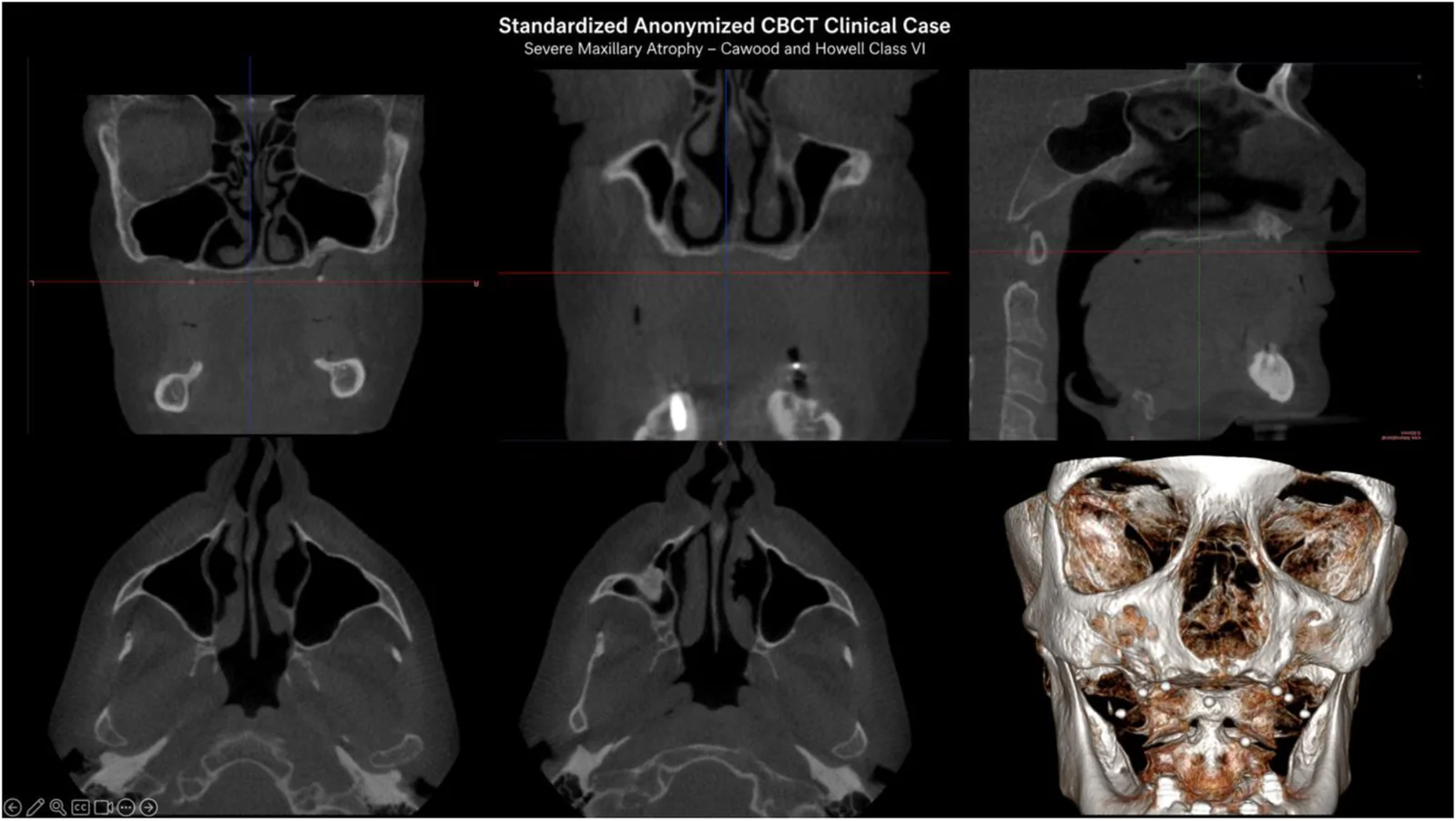
Standardized anonymized CBCT case used for treatment-planning assessment. Representative cone-beam computed tomography (CBCT) images of the standardized clinical case presented to all survey participants. The figure includes coronal sections at different anteroposterior levels, axial sections at different craniocaudal levels, a sagittal reconstruction, and a three-dimensional volume rendering. The images demonstrate severe maxillary atrophy consistent with Cawood and Howell Class VI and served as the radiological basis for therapeutic decision-making within the survey. All patient-identifiable information was removed prior to dissemination.

The questionnaire was structured into three main sections.

The first section collected demographic and professional information, including professional qualification, specialty, workplace setting, geographic area of practice, years of clinical experience, and number of complex rehabilitations performed per year.

The second section presented the standardized clinical case and asked participants to select their preferred therapeutic approach among the following options: alternative implant-based strategies, including zygomatic implants, pterygoid implants, transnasal implants, tilted implants, and patient-specific subperiosteal implants; regenerative approaches with bone grafting followed by conventional implant placement; or hybrid/combined strategies.

The third section explored clinician-reported expectations associated with the selected rehabilitation strategy. Using an internal branching structure, respondents accessed a set of questions specifically related to the treatment approach they had selected. This design allowed clinician-reported expectations and perceptions to be analyzed according to the preferred rehabilitation strategy.

The investigated domains included perceived safety, predictability, tolerability, possibility of immediate loading, expected implant survival at five years, treatment duration, perceived aesthetic outcomes, masticatory function, oral hygiene and maintainability, postoperative discomfort, treatment dropout due to morbidity, annual maintenance costs, clinician-reported patient satisfaction, and perceived quality-of-life improvement.

All questions were formulated as closed-ended items with predefined multiple-choice responses to ensure response standardization and comparability. The full survey questionnaire, including the branching structure according to the selected therapeutic strategy, is provided as Supplementary Material 1.

### 2.4 Questionnaire review and validation

Before international dissemination, the questionnaire underwent internal qualitative review by experienced clinicians involved in maxillofacial surgery, oral surgery, implantology, and prosthetic rehabilitation.

The review process aimed to assess clinical relevance, semantic clarity, logical coherence, and completeness of the investigated domains. Minor wording and structural refinements were introduced before final dissemination.

Formal psychometric validation, including construct validity assessment, factor analysis, or test–retest reliability analysis, was not performed. This was considered appropriate because the questionnaire was designed to investigate explicit clinician-reported expectations and perceptions within a standardized clinical scenario rather than to measure latent psychological constructs.

### 2.5 Data collection procedure

Data were collected through an anonymous online questionnaire hosted on Google Forms and accessible through a direct electronic link.

The survey remained open between July 2025 and November 2025.

The dissemination strategy included direct invitations to clinicians with experience in severe maxillary atrophy rehabilitation, distribution through professional and academic networks, and circulation through scientific mailing lists and international professional societies.

Responses were exported in Microsoft Excel format and screened for eligibility, completeness, and consistency before analysis.

A total of 178 questionnaires were submitted. After exclusion of 23 respondents who did not meet the predefined self-reported experience threshold of at least five complex rehabilitations per year, 155 eligible responses from 33 countries were included in the final analysis, as shown in Figure 2.

**Figure 2:**
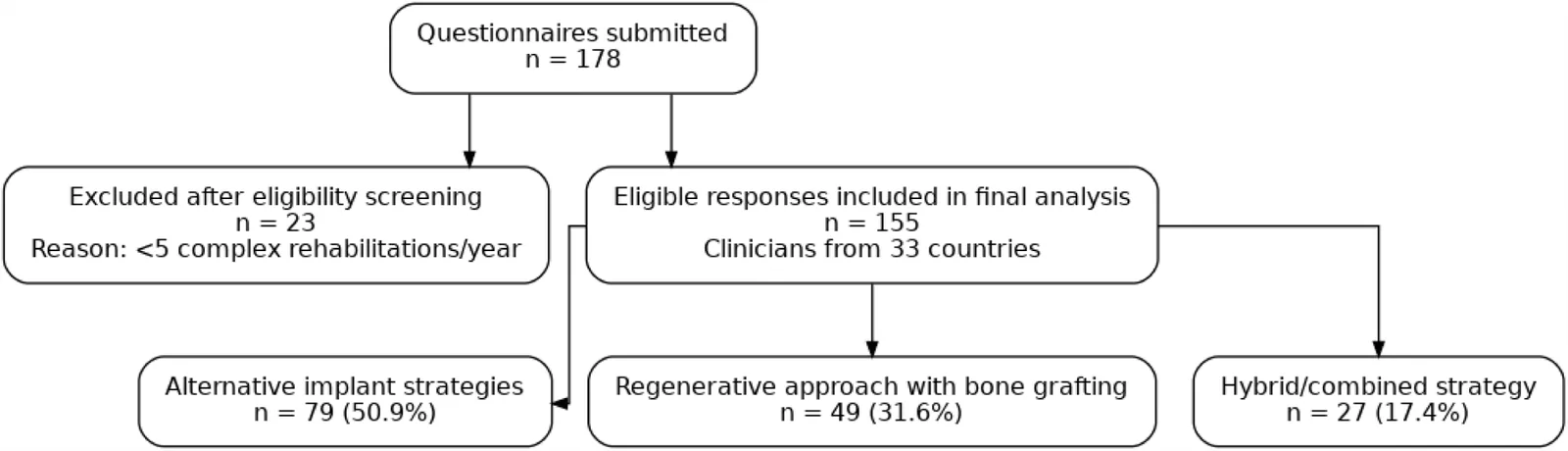
Flowchart of participant selection and therapeutic preference allocation. A total of 178 questionnaires were submitted. After application of the predefined eligibility criterion requiring self-reported experience in at least five complex implant-prosthetic rehabilitations per year, 23 questionnaires were excluded. The final analysis included 155 eligible clinicians from 33 countries. Participants were allocated into three therapeutic preference groups according to the treatment strategy selected for the standardized CBCT case: alternative implant strategies (*n* = 83, 53.5%), regenerative approaches with bone grafting (*n* = 45, 29.0%), and hybrid/combined strategies (*n* = 27, 17.4%).

### 2.6 Outcomes

The primary outcome of the present study was the description of clinician-reported expectations associated with different rehabilitation strategies for a standardized case of severe maxillary atrophy.

Secondary outcomes included clinician-reported perceptions regarding safety, predictability, tolerability, possibility of immediate loading, expected five-year implant survival, overall treatment duration, perceived aesthetic outcomes, smile satisfaction, masticatory function, oral hygiene and maintainability, postoperative discomfort, treatment dropout attributable to morbidity, annual maintenance costs, patient satisfaction, and perceived quality-of-life improvement.

All outcomes were derived from clinician-reported expectations and professional experience associated with the rehabilitation strategy selected by each respondent after evaluation of the standardized CBCT case. The questionnaire was not designed to collect direct clinical outcomes, prospective follow-up data, validated patient-reported outcome measures, or objective measurements of treatment performance.

Accordingly, the findings should be interpreted as clinician-reported expectations and perceptions regarding different rehabilitation strategies rather than as evidence of comparative clinical effectiveness or superiority of any specific treatment approach.

### 2.7 Statistical analysis

The statistical analysis was descriptive in nature.

Categorical variables were summarized using absolute frequencies and percentages.

Percentages were calculated based on the number of valid responses for each survey item. Because the questionnaire employed a branching structure, respondents answered only the questions related to their selected rehabilitation strategy. Consequently, denominators varied according to the therapeutic preference group and are reported where appropriate.

No inferential statistical tests, hypothesis-testing procedures, or multivariable analyses were performed. The aim of the present study was to describe clinician-reported expectations and perceptions associated with different rehabilitation strategies rather than to establish statistical differences between treatment approaches.

Data management and descriptive analyses were performed using Microsoft Excel.

### 2.8 Ethical considerations and data management

Responses were collected anonymously and analyzed in aggregated form.

No patient-identifiable data, human images, or direct patient-reported outcomes were collected. The standardized clinical case used in the survey was anonymized before dissemination.

Participation was voluntary, and completion of the questionnaire was considered as implied consent to participate.

Because the study involved an anonymous survey of healthcare professionals, did not collect identifiable personal data, and did not involve patient recruitment or clinical interventions, formal Institutional Review Board or Ethics Committee approval was not considered necessary according to applicable institutional and international recommendations.

The study was conducted in accordance with the principles of the Declaration of Helsinki.

Data were exported in Microsoft Excel format and stored on password-protected devices in compliance with applicable data protection regulations.

### 2.9 Potential sources of bias

Several limitations inherent to international expert-based surveys should be acknowledged.

Selection bias may have occurred because recruitment was performed through academic, scientific, and professional networks, potentially leading to overrepresentation of highly experienced clinicians, university-affiliated professionals, and high-volume referral centers.

Participation was voluntary, introducing the possibility of self-selection bias, with increased participation from clinicians particularly interested in advanced implant rehabilitation and severe maxillary atrophy management.

The predefined eligibility threshold of at least five complex rehabilitations per year strengthened the expert-based nature of the sample but may have further increased the representation of high-volume clinicians.

In addition, the survey was designed to collect clinician-reported expectations and perceptions rather than direct clinical outcomes. Consequently, responses reflect professional experience, individual beliefs, and anticipated treatment characteristics associated with the selected rehabilitation strategy and should not be interpreted as objective measurements of treatment performance.

An additional limitation relates to the branching structure of the questionnaire. Because respondents evaluated only the rehabilitation strategy they selected as their preferred approach for the standardized clinical case, the findings reflect expectations associated with the chosen strategy rather than direct comparative assessments of multiple treatment modalities performed by the same respondents.

Despite these limitations, the inclusion of experienced clinicians from 33 countries and different professional backgrounds provides a broad overview of contemporary clinician-reported expectations and perceptions regarding the rehabilitation of severe maxillary atrophy.

## 3 Results

### 3.1 Sample characteristics

A total of 178 questionnaires were submitted. After application of the predefined eligibility criterion requiring self-reported experience in at least five complex implant-prosthetic rehabilitations per year, 23 questionnaires were excluded. The final analysis therefore included 155 eligible clinicians.

Eligible responses were obtained from clinicians practicing in 33 countries across Europe, North America, South America, Asia, Africa, and Oceania, supporting the international nature of the survey.

According to the rehabilitation strategy selected for the standardized anonymized CBCT case, participants were allocated into three therapeutic preference groups:

- Alternative implant-based strategies: 83 participants (53.5%)
- Regenerative approaches with bone grafting: 45 participants (29.0%)
- Hybrid/combined strategies: 27 participants (17.4%)

The participant selection process and allocation into therapeutic preference groups are summarized in Figure 2.

### 3.2 Therapeutic preferences

Alternative implant-based strategies represented the most frequently selected rehabilitation approach for the standardized case of severe maxillary atrophy, accounting for 83 of 155 responses (53.5%).

Regenerative approaches with bone grafting were selected by 45 respondents (29.0%), while hybrid or combined strategies were selected by 27 respondents (17.4%).

Overall, these findings indicate that alternative implant-based rehabilitation strategies currently represent the most frequently preferred therapeutic option among experienced clinicians confronted with the same standardized clinical scenario. Nevertheless, regenerative and hybrid approaches continue to account for a substantial proportion of responses, reflecting the coexistence of different therapeutic philosophies in the contemporary management of severe maxillary atrophy.

### 3.3 Perceived safety, predictability, and tolerability

Clinician-reported expectations regarding safety, predictability, and tolerability differed among the three therapeutic preference groups.

In the alternative implant-based strategies group, 67 of 83 respondents (80.7%) considered the selected rehabilitation strategy more predictable, 50 (60.2%) considered it well tolerated by patients, and 46 (55.4%) considered it safer.

In the regenerative approaches group, 32 of 45 respondents (71.1%) considered the selected strategy more predictable, 21 (46.7%) considered it safer, and 15 (33.3%) considered it well tolerated.

In the hybrid approaches group, 11 of 27 respondents (40.7%) considered the selected strategy well tolerated, while 8 respondents (29.6%) considered it more predictable and 8 (29.6%) considered it safer (Table 1).

**Table 1:** Reasons supporting the preferred treatment strategy among respondents.

Treatment strategy	Respondents, *n*	More predictable	Safer	Well tolerated
Alternative implant strategies	83	67 (80.7)	46 (55.4)	50 (60.2)
Regenerative approaches with bone grafting	45	32 (71.1)	21 (46.7)	15 (33.3)
Hybrid/combined strategies	27	8 (29.6)	8 (29.6)	11 (40.7)

Overall, expectations regarding predictability were favorable across all therapeutic preference groups, although they were more frequently reported among respondents selecting alternative implant-based and regenerative approaches. Perceived safety and tolerability showed greater variability, particularly among respondents selecting hybrid strategies.

These findings reflect clinician-reported expectations associated with the selected rehabilitation strategy and should not be interpreted as direct measurements of clinical outcomes or comparative treatment performance.

### 3.4 Immediate loading and treatment duration

Clinician-reported expectations regarding the possibility of immediate loading and overall treatment duration differed substantially among the three therapeutic preference groups.

In the alternative implant-based strategies group, 68 of 83 respondents (81.9%) reported that immediate loading with a fixed prosthesis is almost always possible, 13 (15.7%) considered it feasible only in selected cases, and 2 (2.4%) stated that it is not routinely planned.

In the regenerative approaches group, 21 of 45 respondents (46.7%) indicated that immediate loading is not planned, 22 (48.9%) considered it feasible only in selected cases, and only 2 (4.4%) reported that it is generally applicable.

In the hybrid approaches group, 15 of 27 respondents (55.6%) indicated that immediate loading is feasible only in selected cases, 7 (25.9%) reported that it is generally applicable, and 5 (18.5%) stated that it is not routinely planned (Table 2).

**Table 2:** Feasibility of immediate loading according to preferred treatment strategy.

Treatment strategy	*n*	Immediate loading routinely feasible	Feasible only in selected cases	Not routinely planned
Alternative implant strategies	83	68 (81.9%)	13 (15.7%)	2 (2.4%)
Regenerative approaches with bone grafting	45	2 (4.4%)	22 (48.9%)	21 (46.7%)
Hybrid/combined strategies	27	7 (25.9%)	15 (55.6%)	5 (18.5%)

Expectations regarding overall treatment duration also differed among the three groups. In the alternative implant-based strategies group, 69 of 83 respondents (83.1%) expected functional loading within 72 h, 11 (13.3%) expected treatment completion within 3–6 months, and 3 (3.6%) expected an overall treatment duration of approximately one year.

In the regenerative approaches group, 29 of 45 respondents (64.4%) expected treatment duration of approximately one year, 14 (31.1%) expected treatment completion within 3–6 months, and only 2 (4.4%) expected functional loading within 72 h.

In the hybrid approaches group, 18 of 27 respondents (66.7%) expected treatment completion within 3–6 months, 6 (22.2%) expected treatment duration of approximately one year, and 3 (11.1%) expected functional loading within 72 h (Table 3).

**Table 3:** Estimated treatment duration according to preferred treatment strategy.

Treatment strategy	*n*	Within 72 h	3–6 months	∼1 year
Alternative implant strategies	83	69 (83.1%)	11 (13.3%)	3 (3.6%)
Regenerative approaches with bone grafting	45	2 (4.4%)	14 (31.1%)	29 (64.4%)
Hybrid/combined strategies	27	3 (11.1%)	18 (66.7%)	6 (22.2%)

Overall, respondents selecting alternative implant-based strategies more frequently expected immediate loading and shorter treatment duration, whereas respondents selecting regenerative approaches more commonly anticipated delayed loading protocols and longer treatment times. Hybrid approaches were most frequently associated with intermediate treatment durations and selective use of immediate loading protocols.

### 3.5 Expected five-year implant survival

Clinician-reported expectations regarding five-year implant survival were generally favorable across all therapeutic preference groups, although the distribution of responses differed.

In the alternative implant-based strategies group, 48 of 83 respondents (57.8%) expected implant survival above 95%, 33 (39.8%) expected survival between 85% and 94%, and 2 (2.4%) expected survival below 85%.

In the regenerative approaches group, 18 of 45 respondents (40.0%) expected implant survival above 95%, 26 (57.8%) expected survival between 85% and 94%, and 1 respondent (2.2%) expected survival below 85%.

In the hybrid approaches group, 5 of 27 respondents (18.5%) expected implant survival above 95%, 20 (74.1%) expected survival between 85% and 94%, and 2 respondents (7.4%) expected survival below 85% (Table 4).

**Table 4:** Estimated implant survival according to preferred treatment strategy.

Treatment strategy	*n*	>95%	85%–94%	<85%
Alternative implant strategies	83	48 (57.8%)	33 (39.8%)	2 (2.4%)
Regenerative approaches with bone grafting	45	18 (40.0%)	26 (57.8%)	1 (2.2%)
Hybrid/combined strategies	27	5 (18.5%)	20 (74.1%)	2 (7.4%)

Overall, expectations regarding five-year implant survival were positive across all therapeutic preference groups. Respondents selecting alternative implant-based strategies more frequently anticipated survival rates above 95%, whereas respondents selecting regenerative and hybrid approaches more commonly expected survival rates within the 85%–94% range.

These findings reflect clinician-reported expectations associated with the selected rehabilitation strategy and should not be interpreted as prospective clinical survival data or evidence of comparative treatment effectiveness.

### 3.6 Aesthetic expectations and smile satisfaction

Clinician-reported expectations regarding smile satisfaction were generally favorable across all therapeutic preference groups, although some differences emerged.

In the alternative implant-based strategies group, 69 of 83 respondents (83.1%) expected patients to be satisfied with the appearance of their smile in most cases, while 14 respondents (16.9%) considered satisfaction dependent on limited gingival exposure.

In the regenerative approaches group, 39 of 45 respondents (86.7%) expected patients to be satisfied with the appearance of their smile in most cases, whereas 6 respondents (13.3%) considered satisfaction dependent on the degree of gingival exposure.

In the hybrid approaches group, 15 of 27 respondents (55.6%) expected patients to be satisfied with the appearance of their smile in most cases, 11 respondents (40.7%) considered satisfaction dependent on limited gingival exposure, and 1 respondent (3.7%) expected that additional aesthetic refinements might be required (Table 5).

**Table 5:** Smile aesthetics.

Treatment strategy	*n*	Satisfied	Dependent on gingival exposure	Additional refinements
Alternative implant strategies	83	69 (83.1%)	14 (16.9%)	0 (0.0%)
Regenerative approaches with bone grafting	45	39 (86.7%)	6 (13.3%)	0 (0.0%)
Hybrid/combined strategies	27	15 (55.6%)	11 (40.7%)	1 (3.7%)

Overall, clinician-reported expectations regarding smile aesthetics were favorable across all therapeutic preference groups. Respondents selecting regenerative approaches most frequently anticipated satisfactory smile aesthetics, closely followed by respondents selecting alternative implant-based strategies, whereas expectations appeared more variable among respondents selecting hybrid approaches.

These findings reflect clinician-reported expectations associated with the selected rehabilitation strategy and should not be interpreted as objective measurements of aesthetic outcomes.

### 3.7 Functional expectations

Clinician-reported expectations regarding masticatory function were generally favorable across all therapeutic preference groups.

In the alternative implant-based strategies group, 53 of 83 respondents (63.9%) expected chewing function to be almost comparable to natural teeth with only minor limitations, while 28 respondents (33.7%) expected chewing function to be completely comparable to natural dentition. Only 2 respondents (2.4%) anticipated considerable functional differences.

In the regenerative approaches group, 38 of 45 respondents (84.4%) expected chewing function to be almost comparable to natural teeth with minor limitations, while 7 respondents (15.6%) expected complete comparability with natural dentition. No respondents anticipated considerable functional differences.

In the hybrid approaches group, 14 of 27 respondents (51.9%) expected chewing function to be almost comparable to natural teeth with minor limitations, 10 respondents (37.0%) expected complete comparability with natural dentition, and 3 respondents (11.1%) anticipated considerable functional differences (Table 6).

**Table 6:** Chewing function.

Treatment strategy	*n*	Almost comparable	Completely comparable	Considerable differences
Alternative implant strategies	83	53 (63.9%)	28 (33.7%)	2 (2.4%)
Regenerative approaches with bone grafting	45	38 (84.4%)	7 (15.6%)	0 (0.0%)
Hybrid/combined strategies	27	14 (51.9%)	10 (37.0%)	3 (11.1%)

Overall, clinician-reported expectations regarding masticatory function were favorable across all therapeutic preference groups. Most respondents anticipated complete or near-complete restoration of chewing ability following rehabilitation, regardless of the selected treatment strategy.

These findings reflect clinician-reported expectations associated with the selected rehabilitation strategy and should not be interpreted as direct measurements of functional outcomes.

### 3.8 Oral hygiene and maintainability

Clinician-reported expectations regarding oral hygiene and maintainability were generally favorable across all therapeutic preference groups, although some differences emerged.

In the alternative implant-based strategies group, 54 of 83 respondents (65.1%) expected hygiene-related difficulties to be limited to the initial adaptation period, 26 respondents (31.3%) anticipated that patients would frequently require special hygiene tools or additional maintenance measures, and 3 respondents (3.6%) expected oral hygiene to be as simple as with natural dentition.

In the regenerative approaches group, 29 of 45 respondents (64.4%) expected hygiene-related difficulties to be limited to the initial adaptation period, 9 respondents (20.0%) anticipated frequent need for special hygiene measures, and 7 respondents (15.6%) expected oral hygiene to be comparable to that of natural teeth.

In the hybrid approaches group, 16 of 27 respondents (59.3%) expected hygiene-related difficulties to be mainly confined to the initial adaptation period, 8 respondents (29.6%) anticipated frequent need for special hygiene measures, and 3 respondents (11.1%) expected oral hygiene to be comparable to that of natural teeth (Table 7).

**Table 7:** Oral hygiene management.

Treatment strategy	*n*	Adaptation then stable	More demanding	No substantial differences
Alternative implant strategies	83	53 (63.9%)	20 (24.1%)	10 (12.0%)
Regenerative approaches with bone grafting	45	30 (66.7%)	10 (22.2%)	5 (11.1%)
Hybrid/combined strategies	27	17 (63.0%)	7 (25.9%)	3 (11.1%)

Overall, most respondents across all therapeutic preference groups expected oral hygiene challenges to be concentrated during the initial adaptation phase and to become more manageable over time. Expectations regarding long-term maintainability were generally favorable, although a proportion of respondents anticipated the need for additional hygiene measures and patient education.

These findings reflect clinician-reported expectations associated with the selected rehabilitation strategy and should not be interpreted as direct measurements of maintenance requirements or oral hygiene outcomes.

### 3.9 Annual maintenance cost expectations

Clinician-reported expectations regarding annual maintenance costs were generally favorable across all therapeutic preference groups, although some differences emerged.

In the alternative implant-based strategies group, 55 of 83 respondents (66.3%) considered annual maintenance costs comparable to those associated with other rehabilitation strategies, 22 respondents (26.5%) indicated that costs depend on individual patient characteristics and clinical circumstances, and 6 respondents (7.2%) anticipated higher maintenance costs due to the need for more frequent follow-up visits.

In the regenerative approaches group, 36 of 45 respondents (80.0%) considered annual maintenance costs comparable to those of other rehabilitation strategies, while 9 respondents (20.0%) indicated that maintenance requirements depend on patient-specific factors. No respondents in this group consistently anticipated higher maintenance costs.

In the hybrid approaches group, 16 of 27 respondents (59.3%) considered annual maintenance costs comparable to those associated with other rehabilitation strategies, 7 respondents (25.9%) indicated that costs depend on individual patient characteristics, and 4 respondents (14.8%) anticipated higher maintenance costs due to the need for more frequent maintenance visits (Table 8).

**Table 8:** Maintenance costs.

Treatment strategy	*n*	Comparable	Patient-dependent	Higher
Alternative implant strategies	83	55 (66.3%)	22 (26.5%)	6 (7.2%)
Regenerative approaches with bone grafting	45	36 (80.0%)	9 (20.0%)	0 (0.0%)
Hybrid/combined strategies	27	16 (59.3%)	7 (25.9%)	4 (14.8%)

Overall, most respondents across all therapeutic preference groups expected annual maintenance costs to be broadly comparable to those associated with alternative rehabilitation strategies. However, a proportion of respondents anticipated variability related to patient-specific factors, particularly among alternative implant-based and hybrid approaches.

These findings reflect clinician-reported expectations associated with the selected rehabilitation strategy and should not be interpreted as objective economic evaluations or direct measurements of long-term maintenance costs.

### 3.10 Patient satisfaction and quality-of-life expectations

Clinician-reported expectations regarding patient satisfaction and quality-of-life improvement were generally favorable across all therapeutic preference groups.

In the alternative implant-based strategies group, 66 of 83 respondents (79.5%) expected very high patient satisfaction (>90% of patients satisfied) one year after prosthetic loading, while 16 respondents (19.3%) expected moderate satisfaction levels. One respondent (1.2%) reported insufficient data to estimate patient satisfaction. Regarding quality of life, 77 respondents (92.8%) expected a significant improvement following treatment, 5 (6.0%) expected a moderate improvement, and 1 respondent (1.2%) expected no noticeable change.

In the regenerative approaches group, 37 of 45 respondents (82.2%) expected very high patient satisfaction, while 6 respondents (13.3%) expected moderate satisfaction levels. Two respondents (4.4%) reported insufficient data to estimate patient satisfaction. Regarding quality of life, 44 respondents (97.8%) expected a significant improvement, while 1 respondent (2.2%) expected a moderate improvement.

In the hybrid approaches group, 21 of 27 respondents (77.8%) expected very high patient satisfaction, while 6 respondents (22.2%) expected moderate satisfaction levels. Similarly, 21 respondents (77.8%) expected a significant improvement in quality of life, whereas 6 respondents (22.2%) expected a moderate improvement (Tables 9, 10).

**Table 9:** Patient satisfaction.

Treatment strategy	*n*	Very high	Moderate	Insufficient data
Alternative implant strategies	83	66 (79.5%)	16 (19.3%)	1 (1.2%)
Regenerative approaches with bone grafting	45	37 (82.2%)	6 (13.3%)	2 (4.4%)
Hybrid/combined strategies	27	21 (77.8%)	6 (22.2%)	0 (0.0%)

**Table 10:** Quality of life.

Treatment strategy	*n*	Significant improvement	Moderate improvement	No improvement
Alternative implant strategies	83	77 (92.8%)	5 (6.0%)	1 (1.2%)
Regenerative approaches with bone grafting	45	44 (97.8%)	1 (2.2%)	0 (0.0%)
Hybrid/combined strategies	27	21 (77.8%)	6 (22.2%)	0 (0.0%)

Overall, respondents across all therapeutic preference groups anticipated high levels of patient satisfaction and substantial improvements in quality of life following rehabilitation. Expectations were particularly favorable among respondents selecting alternative implant-based and regenerative approaches, although positive expectations were reported across all groups.

These findings reflect clinician-reported expectations associated with the selected rehabilitation strategy and should not be interpreted as direct patient-reported outcomes or validated quality-of-life measurements.

### 3.11 Postoperative morbidity expectations

Clinician-reported expectations regarding postoperative morbidity differed among the three therapeutic preference groups.

In the alternative implant-based strategies group, 35 of 83 respondents (42.2%) expected postoperative discomfort to last less than one week, 43 (51.8%) expected discomfort lasting between one and four weeks, and 5 respondents (6.0%) expected symptoms to persist for approximately one month.

In the regenerative approaches group, 14 of 45 respondents (31.1%) expected postoperative discomfort to last less than one week, 26 (57.8%) expected discomfort lasting between one and four weeks, and 5 respondents (11.1%) expected symptoms to persist for approximately one month.

In the hybrid approaches group, 6 of 27 respondents (22.2%) expected postoperative discomfort to last less than one week, 17 (63.0%) expected discomfort lasting between one and four weeks, and 4 respondents (14.8%) expected symptoms to persist for approximately one month (Table 11).

**Table 11:** Postoperative discomfort.

Treatment strategy	*n*	<1 week	1–4 weeks	>1 month
Alternative implant strategies	83	35 (42.2%)	39 (47.0%)	9 (10.8%)
Regenerative approaches with bone grafting	45	7 (15.6%)	26 (57.8%)	12 (26.7%)
Hybrid/combined strategies	27	5 (18.5%)	15 (55.6%)	7 (25.9%)

Expectations regarding treatment abandonment due to morbidity were generally favorable across all therapeutic preference groups. In the alternative implant-based strategies group, 67 of 83 respondents (80.7%) expected treatment abandonment rates below 5%, while 14 (16.9%) expected rates between 5% and 15%, and 2 respondents (2.4%) expected rates above 15%.

In the regenerative approaches group, 33 of 45 respondents (73.3%) expected treatment abandonment rates below 5%, 11 (24.4%) expected rates between 5% and 15%, and 1 respondent (2.2%) expected rates above 15%.

In the hybrid approaches group, 18 of 27 respondents (66.7%) expected treatment abandonment rates below 5%, while 9 respondents (33.3%) expected rates between 5% and 15%. No respondents expected abandonment rates above 15% (Table 12).

**Table 12:** Dropout.

Treatment strategy	*n*	<5%	5%–15%	>15%
Alternative implant strategies	83	67 (80.7%)	14 (16.9%)	2 (2.4%)
Regenerative approaches with bone grafting	45	33 (73.3%)	11 (24.4%)	1 (2.2%)
Hybrid/combined strategies	27	18 (66.7%)	9 (33.3%)	0 (0.0%)

Overall, most respondents across all therapeutic preference groups anticipated postoperative discomfort lasting between one and four weeks and expected treatment abandonment attributable to morbidity to remain below 5% in the majority of cases.

These findings reflect clinician-reported expectations associated with the selected rehabilitation strategy and should not be interpreted as direct measurements of postoperative morbidity or treatment adherence.

## 4 Discussion

### 4.1 Principal findings

The present international cross-sectional survey explored clinician-reported expectations associated with different rehabilitation strategies for severe maxillary atrophy using a standardized anonymized Cawood and Howell Class VI case.

The most important finding of this study is that clinicians selecting different therapeutic approaches reported different expectations regarding treatment characteristics and anticipated outcomes. Alternative implant-based strategies were more frequently associated with expectations of immediate loading, shorter treatment duration, high predictability, and favorable implant survival. In contrast, clinicians selecting regenerative approaches more commonly anticipated longer treatment pathways but also reported favorable expectations regarding smile satisfaction, functional rehabilitation, and quality-of-life improvement. Hybrid approaches generally occupied an intermediate position and were characterized by greater variability across most investigated domains.

Importantly, despite these differences, clinician-reported expectations were generally positive across all therapeutic preference groups. High levels of anticipated patient satisfaction, substantial improvements in quality of life, favorable masticatory function, and acceptable long-term maintenance requirements were consistently reported regardless of the selected rehabilitation strategy.

These findings suggest that contemporary management of severe maxillary atrophy is increasingly influenced by different treatment philosophies and clinical priorities rather than by a universally accepted therapeutic hierarchy. Clinicians appear to balance treatment duration, surgical complexity, prosthetic objectives, anticipated morbidity, aesthetic considerations, and patient-related factors when selecting a rehabilitation strategy.

The results should be interpreted within the context of clinician-reported perceptions and expectations rather than objective clinical outcomes. Consequently, the present study does not provide evidence of superiority of any specific rehabilitation approach but instead offers insight into how experienced clinicians anticipate the potential benefits and limitations of different treatment strategies when evaluating the same complex clinical scenario.

### 4.2 Expectations regarding immediate loading and treatment duration

One of the most consistent findings of the present survey was the difference in clinician-reported expectations regarding treatment timing. Respondents selecting alternative implant-based strategies more frequently anticipated immediate loading and rehabilitation within a very short timeframe, whereas respondents selecting regenerative approaches generally expected longer treatment pathways extending over several months or up to one year. Clinicians selecting hybrid approaches reported intermediate expectations.

These differences likely reflect the distinct biological and therapeutic objectives associated with the selected rehabilitation strategies. Clinicians who favor alternative implant-based approaches may place greater emphasis on reducing treatment duration and restoring function as early as possible. This perception is consistent with the rationale of graftless rehabilitation concepts, which aim to exploit residual basal bone or remote skeletal anchorage sites while avoiding the healing periods typically associated with bone augmentation procedures (5, 12–15).

Conversely, clinicians selecting regenerative approaches may accept longer treatment times as an inherent component of a reconstructive pathway designed to restore bone volume and create favorable conditions for implant placement. Previous studies have described bone grafting and guided bone regeneration as staged procedures requiring graft maturation, implant osseointegration, and subsequent prosthetic rehabilitation before definitive functional loading can be achieved (1–4).

Interestingly, the expectations reported by respondents appear to reflect broader trends in contemporary implant rehabilitation. Rather than representing competing philosophies, graftless and regenerative approaches may be viewed as alternative strategies designed to address different clinical priorities and patient needs. Consequently, treatment duration may be perceived not simply as a limitation but as a characteristic intrinsically linked to the biological objectives of the selected rehabilitation pathway (16–18).

Importantly, these findings should not be interpreted as evidence that one treatment strategy provides superior clinical outcomes compared with another. Instead, they illustrate how clinicians selecting different rehabilitation approaches anticipate treatment timing and loading protocols when confronted with the same severe maxillary atrophy scenario.

### 4.3 Expectations regarding predictability and implant survival

Another important finding of the present survey concerns clinician-reported expectations regarding predictability and long-term implant survival. Across all therapeutic preference groups, most respondents anticipated favorable outcomes, although clinicians selecting alternative implant-based strategies more frequently reported expectations of very high implant survival and greater predictability.

These findings likely reflect the increasing confidence that many clinicians have developed toward contemporary graftless rehabilitation concepts. Over the last two decades, growing clinical experience and an expanding body of evidence have supported the use of zygomatic implants, pterygoid implants, and other remote anchorage solutions for the rehabilitation of severely atrophic maxillae. As a result, clinicians who routinely adopt these approaches may perceive them as highly reliable and predictable treatment options (5, 12, 14, 15).

Similarly, the favorable expectations reported by respondents selecting patient-specific implant solutions may reflect recent technological advances in digital planning, CAD-CAM manufacturing, and personalized implant design. Contemporary subperiosteal implant systems differ substantially from historical designs and have been associated with encouraging medium-term outcomes in selected patient populations (6, 16, 19).

Importantly, clinicians selecting regenerative approaches also reported highly favorable expectations regarding implant survival and predictability. This observation is consistent with the long-standing role of bone grafting and guided bone regeneration as established reconstructive strategies for severe alveolar deficiencies. Despite requiring additional surgical stages and longer treatment times, these procedures continue to be widely regarded as reliable approaches capable of supporting long-term implant rehabilitation (1–4).

The slightly more conservative expectations observed among respondents selecting hybrid approaches may reflect the complexity and heterogeneity of these treatment concepts. Because hybrid rehabilitation frequently combines different biological and prosthetic principles, clinicians may perceive outcomes as being more dependent on individual anatomical and clinical circumstances.

Importantly, the present findings should not be interpreted as evidence of actual implant survival or comparative treatment effectiveness. Rather, they reflect clinician perceptions regarding the reliability and long-term performance of different rehabilitation strategies when applied to the same severe maxillary atrophy scenario. The generally favorable expectations observed across all groups suggest that multiple therapeutic pathways are currently perceived as predictable options when appropriate patient selection, treatment planning, and clinical execution are achieved.

### 4.4 Aesthetic expectations and smile satisfaction

Aesthetic rehabilitation represents a key objective in the treatment of severe maxillary atrophy and is often considered alongside functional recovery when evaluating treatment success. In the present survey, clinician-reported expectations regarding smile satisfaction were generally favorable across all therapeutic preference groups, although some differences emerged in the degree of confidence expressed by respondents.

Clinicians selecting regenerative approaches most frequently anticipated high levels of smile satisfaction. This perception may reflect the reconstructive philosophy underlying bone augmentation procedures, which aim not only to facilitate implant placement but also to restore lost alveolar volume and improve soft-tissue support. From the clinician's perspective, the possibility of recreating more favorable anatomical conditions may contribute to expectations of improved aesthetic integration and smile appearance (1–4).

At the same time, respondents selecting alternative implant-based strategies also reported highly favorable expectations regarding smile satisfaction. This finding suggests that many clinicians perceive contemporary graftless rehabilitation concepts as capable of achieving satisfactory aesthetic outcomes despite the absence of extensive reconstructive procedures. Advances in digital treatment planning, prosthetic design, and patient-specific implant technologies may have contributed to increasing confidence in the aesthetic potential of these approaches (6, 7, 16, 17).

The greater variability observed among clinicians selecting hybrid approaches may reflect the heterogeneous nature of these treatment concepts. Because hybrid rehabilitation frequently combines reconstructive and graftless elements, aesthetic expectations may be influenced by a broader range of anatomical, prosthetic, and patient-related factors (20).

Importantly, the present findings should not be interpreted as evidence of actual aesthetic outcomes. Rather, they reflect clinician expectations regarding the likelihood of achieving satisfactory smile aesthetics following rehabilitation. Aesthetic success remains a multifactorial concept influenced by soft-tissue characteristics, lip dynamics, prosthetic design, residual anatomy, and individual patient expectations. Consequently, the favorable expectations reported across all groups may indicate that clinicians believe satisfactory aesthetic rehabilitation can be achieved through different therapeutic pathways when treatment planning is appropriately tailored to the individual patient.

### 4.5 Functional expectations, patient satisfaction, and quality of life

The restoration of oral function and the improvement of patient well-being represent central objectives in the rehabilitation of severe maxillary atrophy. In the present survey, clinician-reported expectations regarding masticatory function, patient satisfaction, and quality-of-life improvement were consistently favorable across all therapeutic preference groups.

Most respondents anticipated complete or near-complete restoration of chewing ability following rehabilitation, regardless of the selected treatment strategy. Similarly, expectations regarding patient satisfaction and quality-of-life improvement were overwhelmingly positive, with most clinicians anticipating substantial benefits after treatment.

These findings may reflect a shared perception among experienced clinicians that successful rehabilitation extends beyond implant survival and prosthetic stability alone. Contemporary treatment planning increasingly incorporates broader objectives, including restoration of oral function, improvement of social confidence, enhancement of daily activities, and overall patient well-being (10, 21, 22).

Interestingly, despite the substantial differences among the investigated rehabilitation strategies, clinicians reported similarly favorable expectations regarding patient-centered outcomes. This observation suggests that respondents may perceive different therapeutic pathways as capable of achieving comparable functional and quality-of-life goals, even when based on distinct surgical and biological principles.

Previous studies have highlighted the importance of patient-reported outcome measures in the evaluation of implant-supported rehabilitation of the severely atrophic maxilla. Improvements in oral health-related quality of life, masticatory efficiency, comfort, and overall treatment satisfaction have been reported following both graft-based and graftless rehabilitation protocols (10, 21). Such evidence may contribute to the positive expectations expressed by clinicians in the present survey.

Importantly, the present findings should not be interpreted as evidence of actual patient satisfaction, functional recovery, or quality-of-life improvement. Rather, they reflect clinician expectations regarding the outcomes they anticipate when applying the selected rehabilitation strategy. Future prospective studies comparing clinician expectations with directly measured patient-reported outcomes may help clarify the relationship between perceived and observed treatment benefits.

### 4.6 Oral hygiene, maintenance, and morbidity expectations

In addition to functional and aesthetic outcomes, clinicians also reported expectations regarding long-term maintenance requirements and treatment-related morbidity. Overall, respondents across all therapeutic preference groups anticipated manageable oral hygiene requirements, acceptable maintenance demands, and relatively low rates of treatment discontinuation attributable to morbidity.

Most clinicians expected oral hygiene challenges to be concentrated during the initial adaptation period rather than representing a persistent long-term limitation. This observation may reflect the widespread recognition that patient education, professional maintenance programs, and appropriate prosthetic design play a critical role in the long-term success of implant-supported rehabilitation. Regardless of the selected rehabilitation strategy, respondents generally anticipated that patients would be able to maintain adequate oral hygiene following an initial learning phase.

Expectations regarding annual maintenance costs were similarly favorable. Most clinicians considered maintenance requirements broadly comparable to those associated with alternative rehabilitation strategies, although some respondents anticipated greater variability depending on individual patient characteristics, prosthetic design, and the complexity of the rehabilitation. These findings suggest that long-term maintenance may be perceived as an integral component of implant rehabilitation rather than a major limitation of any specific treatment approach.

Clinician-reported expectations regarding postoperative morbidity also demonstrated interesting patterns. Although most respondents anticipated postoperative discomfort lasting between one and four weeks, treatment abandonment attributable to morbidity was generally expected to remain below 5% across all therapeutic preference groups. These findings indicate that clinicians perceive the investigated rehabilitation strategies as generally acceptable from a patient tolerance perspective, despite differences in surgical complexity and treatment duration.

The relatively favorable expectations regarding morbidity may reflect advances in surgical planning, digital technologies, perioperative management, and patient selection that have occurred across both reconstructive and graftless rehabilitation protocols in recent years. Contemporary evidence suggests that improvements in surgical techniques and rehabilitation protocols have contributed to reducing treatment burden and improving overall patient acceptance, although procedure-specific complications and maintenance requirements remain important considerations (3, 8, 23–28).

Importantly, the present findings should not be interpreted as direct measurements of maintenance requirements, patient compliance, postoperative discomfort, or treatment adherence. Rather, they represent clinician expectations regarding factors that may influence long-term treatment success and patient acceptance. Future prospective investigations incorporating patient-reported outcomes and objective maintenance data may help determine the extent to which these expectations correspond to real-world clinical experience.

### 4.7 Clinical implications

The findings of the present study may have several clinical implications for the management of severe maxillary atrophy. While numerous studies have focused on clinical outcomes associated with individual rehabilitation techniques, comparatively little attention has been devoted to understanding how clinicians perceive and anticipate the potential advantages and limitations of different treatment strategies when evaluating the same complex clinical scenario.

The results suggest that clinicians selecting different rehabilitation approaches often develop distinct expectations regarding treatment duration, predictability, functional recovery, aesthetic outcomes, maintenance requirements, and patient-centered benefits. These expectations may influence treatment planning, patient counseling, informed consent discussions, and ultimately therapeutic decision-making.

Importantly, the present findings highlight that contemporary rehabilitation of severe maxillary atrophy is not characterized by a single universally accepted treatment pathway. Rather, multiple therapeutic strategies are currently perceived as valid options, each associated with specific biological principles, clinical priorities, and anticipated outcomes. This observation is consistent with recent literature emphasizing individualized treatment planning and the need to adapt rehabilitation strategies to patient-specific anatomical, functional, and psychosocial characteristics (8, 18, 19, 29–31).

From a practical perspective, understanding clinician expectations may help identify areas of consensus as well as domains where perceptions differ substantially among therapeutic philosophies. Such information may contribute to improving interdisciplinary communication, facilitating shared decision-making, and guiding future research aimed at comparing anticipated and observed treatment outcomes.

Finally, the distinction between clinician expectations and actual clinical outcomes deserves particular attention. Expectations influence treatment recommendations and patient communication, but they do not necessarily correspond to real-world results. Future studies comparing clinician-reported expectations with prospectively collected clinical and patient-reported outcomes may therefore provide valuable information regarding the accuracy of current therapeutic perceptions and decision-making processes.

### 4.8 Strengths, limitations, and future perspectives

The present study has several strengths. To the authors' knowledge, this is one of the first international surveys specifically designed to investigate clinician-reported expectations associated with different rehabilitation strategies for severe maxillary atrophy using a standardized anonymized Cawood and Howell Class VI case. The use of a single clinical scenario allowed respondents to evaluate the same anatomical conditions, thereby reducing variability related to case complexity and facilitating comparison of clinician perceptions across different therapeutic preference groups.

Another strength is the international composition of the study population, which included experienced clinicians from multiple countries and professional backgrounds involved in the management of complex implant-prosthetic rehabilitation. This diversity provides a broad overview of contemporary perceptions and expectations regarding the treatment of severe maxillary atrophy.

Nevertheless, several limitations should be acknowledged. First, the study was based on clinician-reported expectations rather than direct clinical outcomes. Consequently, the findings should not be interpreted as evidence of actual treatment performance, implant survival, patient satisfaction, or quality-of-life improvement. Instead, they reflect how clinicians anticipate potential outcomes when evaluating a standardized clinical scenario.

Second, the survey design inherently captures subjective perceptions that may be influenced by individual clinical experience, training background, preferred treatment philosophy, geographic practice patterns, and familiarity with specific rehabilitation techniques. As a result, expectations reported by respondents may not necessarily correspond to objectively measured outcomes observed in clinical practice.

Third, although the use of a standardized case improves comparability, real-world treatment planning is often influenced by additional patient-specific factors that could not be fully represented within a survey-based model. Factors such as medical comorbidities, patient preferences, economic considerations, soft-tissue characteristics, and psychosocial aspects may substantially influence therapeutic decision-making in routine clinical practice.

Finally, the cross-sectional nature of the survey precludes any assessment of causal relationships between clinician expectations and actual treatment outcomes. Future prospective investigations comparing clinician-reported expectations with objectively measured clinical outcomes and patient-reported outcome measures may help clarify the relationship between anticipated and observed treatment results. Such studies could provide valuable insight into how expectations influence therapeutic decision-making and whether these expectations accurately reflect real-world clinical performance.

## 5 Conclusions

This international cross-sectional survey provides insight into clinician-reported expectations associated with different rehabilitation strategies for severe maxillary atrophy.

Clinicians selecting alternative implant-based, regenerative, and hybrid approaches reported distinct expectations regarding treatment duration, immediate loading, predictability, implant survival, aesthetic outcomes, maintenance requirements, postoperative morbidity, patient satisfaction, and quality-of-life improvement. These differences appear to reflect diverse therapeutic philosophies and clinical priorities rather than a universally accepted treatment hierarchy.

Despite these differences, expectations were generally favorable across all therapeutic preference groups, suggesting that multiple rehabilitation pathways are currently perceived as viable options for the management of severe maxillary atrophy when appropriate case selection and treatment planning are performed.

Importantly, the findings should be interpreted as clinician-reported expectations rather than direct measurements of clinical outcomes or patient-reported outcomes. Future prospective studies comparing clinician expectations with actual clinical and patient-centered outcomes may help clarify the relationship between anticipated and observed treatment results and further improve decision-making in the rehabilitation of severe maxillary atrophy.

## Data Availability

The raw data supporting the conclusions of this article will be made available by the authors, without undue reservation.

## The Collaborative Working Group

Department of Periodontics and Preventive Dentistry, School of Dental Medicine, University of Pittsburgh, Pittsburgh, Pennsylvania, USA: Martinna Bertolini. Department of Periodontics and Preventive Dentistry, School of Dental Medicine, University of Pittsburgh, Pittsburgh, Pennsylvania, USA: Cristiano Soares Moreira. Department of Oral Medicine, Infection, & Immunity, Harvard School of Dental Medicine: Shayan Barootchi. Private Practice, Fresno CA: Ryan Andrew Dunlop. Department of Orofacial Sciences, School of Dentistry, University of California, San Francisco, USA: Guo-Hao Lin. Department of Oral and Maxillofacial Surgery, University of Arizona PHX Campus, Banner University Medical Center, Phoenix, Arizona, USA: Vishtasb Broumand. Departamento de Cirugia Oral y Maxilofacial Universidad de la Republica Oriental del Uruguay, Hospital Italiano Montevideo Uruguay: Felipe Kornecki Radzinski. Department of Periodontology, School of Dentistry, University of Başkent, Ankara, Turkey: Emine Elif Alaaddinoglu. Department of Oral and Maxillofacial Surgery, Faculty of Dentistry, Recep Tayyip Erdogan University, Rize, Türkiye: Alperen Kalyouncu. Department of Periodontology, Faculty of Dentistry, Gazi University, Ankara, Türkiye: Sila Cagri Isler. Second Dental Center, Shanghai Ninth People's Hospital, Shanghai Jiao Tong University, Shanghai, China: Yiqun Wu. Smart Arches Dental Implant Centers USA: Simon Oh. Department of Oral and Maxillofacial Surgery, Faculty of Dentistry, University of Dicle, Diyarbakır, Turkey: Ridvan Guler. Department of Oral and Maxillofacial Surgery, Faculty of Dentistry, İzmir Katip Çelebi University, İzmir, Türkiye: Onur Şahin. Department of Periodontology, School of Dentistry, University of Başkent, Ankara, Turkey: Engin Ozgur. INNOTERE Biomaterials, Radebeul, Germany: Sascha Heinemann. Department of Oral Health & Medicine, University Center for Dental Medicine Basel (UZB), University of Basel, Basel, Switzerland: Michael Marc Bornstein. Department of Oral Surgery and Stomatology, University of Bern School of Dental Medicine, Bern, Switzerland: Emilio Couso-Queiruga. Department of Oral Surgery and Stomatology, University of Bern School of Dental Medicine, Bern, Switzerland: Clemens Raabe. Perio-Implant Innovation Center, Institute of Integrated Oral, Craniofacial and Sensory Research, Shanghai Ninth's People Hospital, Shanghai Jiao Tong School of Medicine, Shanghai, China: Andrea Roccuzzo. Clinic of Reconstructive Dentistry, Center for Dental Medicine, University of Zurich, Zurich, Switzerland: Daniel Thoma. State Key Laboratory of Oral Diseases, National Center for Stomatology, National Clinical Research Center for Oral Diseases, West China Hospital of Stomatology, Sichuan University, Chengdu, China: Yi Man. Department of Oral Biochemistry, Institute of Odontology, The Sahlgrenska Academy at University of Gothenburg, Gothenburg, Sweden: Farah Asa'ad. Department of Odontology, Umeå University, Sweden: Mats Sjöström. Department of Periodontology, College of Dentistry, Yonsei University, Seoul, South Korea: Jae-Kook Cha., Private Practice, Centre for Implantology and Oral Medicine, Johannesburg, South Africa: Muhammed Hanif Abram. Head of Clinical Department of Maxillofacial Surgery, Russian University of Medicine, Moscow, Russia: Nicolai Redko. Head of Clinical Department of Maxillofacial Surgery, Russian University of Medicine, Moscow, Russia: Alexey Drobyshev. Head of Clinical Department of Maxillofacial Surgery, Russian University of Medicine, Moscow, Russia: Eduard Kharazyan. Private Practice, Florence, Italy: Cesare Paoleschi. Wickham Dental Care, Carseldine QLD, Australia: Sadesh Kumar. Department of Propaedeutics of Dental Diseases, Faculty of Dentistry, Bashkir State Medical University, Ufa, Russia: Andrey Trokhalin., Department of Maxillofacial and Plastic Surgery, B.V. Petrovsky Russian Scientific Center of Surgery, Moscow, Russia: Ilya Bozo. Department of Oral and Maxillofacial Surgery, Fogolyán Kristóf Emergency County Hospital, Sfantu Gheorghe, Romania: Zoltán Fábián. Department of Implantology, Apollonia University Iasi, Iasi, Romania: Dragan Eliza., Victor Babeș University of Medicine and Pharmacy Timișoara, Timișoara, Romania: Mariu Leretter. Department of Oral and Maxillofacial Surgery, Clitrofa-Centro Médico, Dentário e Cirúrgico, Trofa, Portugal: Fernando Manuel Pinto Duarte. Diamante Dental Clinics, Lubin/Zielona Góra, Poland: Adam Aleksnder Nowicki. University of Oslo, Oslo, Norway: Ilaria Vitali. Department of Oral and Maxillofacial Surgery, Treant Hospital Scheper, Emmen, The Netherlands: Jurjen Schortinghuis. Department of Oral and Maxillofacial Surgery, Erasmus MC University Medical Center, Rotterdam, the Netherlands: Justin Pijpe. Department of Oral and Maxillofacial Surgery, University of Groningen, University Medical Center Groningen, Groningen, The Netherlands: Gerry M Raghoebar. Department of Oral and Maxillofacial surgery, Micunovic medical clinic, Podgorica, Montenegro: Miljan Micunovic. Department of Odontology and periodontology, Sofia Sirbu”,USMF “Nicolae Testemitanu”, Chișinău, Moldavia: Viorica Chetrus. Private practice, Meliva VIC klinikos, Vilnius, Lithuania: Algirdas Puisys. Assistant Lecturer of Prosthetic Dentistry, Alasmarya Islamic University, Zliten, Libya: Mohammad Ibrahim Alghraisi. Department of Maxillofacial Surgery, Policlinico di Bari, Bari, Italy: Stefan Cocis. Department of Oral Surgery, School of Dentistry, University of Padova: Rocco Borrello. Department of Oral Surgery, School of Dentistry, University of Padova: Alessandro Paccagnella. Department of Maxillofacial Surgery, University of Siena, Siena, Italy: Lisa Catarzi. Department of Maxillofacial Surgery, University of Siena, Siena, Italy; Division of Maxillofacial Surgery, Marche University Hospitals- Umberto I, Ancona, Italy: Giulio Cirignaco. Department of Dentistry, Catholic University of the Sacred Heart, University of Rome, Rome, Italy: Alessandro Ciolfi. Department of Oral and Maxillofacial Surgery, Aalborg University Hospital, Aalborg, Denmark: Thomas Starch-Jensen. Department of Oral and Maxillofacial Surgery, Aalborg University Hospital, Aalborg, Denmark: Hanna Cecilia Aludden. Department of Oral Surgery, Gabriele d'Annunzio University of Chieti-Pescara, Italy: Paolo Ricci. Department of Oral Surgery, Gabriele d'Annunzio University of Chieti-Pescara, Italy: Stefania Romano. Department of Maxillofacial Surgery Spedali Civili, Brescia, Italy: Stefano Negrini. Integrated Service Maxillofacial Surgery/Head-Neck Surgery, Nefrocenter Healthcare System and Hospital Group, Adjunct Assistant Professor Dept. ENT/Head-Neck Surgery University of Maryland School of Medicine—Baltimore, USA: Vito del Deo. Division of Maxillofacial Surgery, Marche University Hospitals- Umberto I, Ancona, Italy:Giuseppe Consorti. Department of Oral and Maxillofacial Surgery, County Hospital “San Bortolo”, Vicenza, Italy: Riccardo Girotto. Private Practice, Genova, Italy: Roberto Rossi. Private practice, Gasport, NY, USA: Igor Kaplasky. Department of Head and Neck and Neurosciences, Maxillofacial Surgery Unit, San Camillo-Forlanini Hospital, Rome, Italy: Flavio Andrea Govoni. Department of Head and Neck and Neurosciences, Maxillofacial Surgery Unit, San Camillo-Forlanini Hospital, Rome, Italy: Roberto Pistilli. Department of General Sugery and Surgical-Medical Specialties, School of Dentistry, University of Catania, 95123 Catania, Italy: Gaetano Isola. Division of Maxillofacial Surgery, Marche University Hospitals-Umberto I, Ancona, Italy: Andrea Balercia. Department of Oral and Maxillofacial Sciences, Sapienza University of Rome, Italy: Edoardo Brauner. Department of Neuroscience, School of Dentistry, University of Padova, Padova, Italy: Stefano Sivolella. Unit of Oral and Maxillofacial Surgery and Odontostomatology, University of Verona, Verona, Italy: Gianluca Colapinto. Department of Oral and Maxillofacial Surgery, School of Dentistry, Internation University of Catalonia, Barcelona, Spain: Adaia Valls-Ontañón. Department of Oral surgery and implantology, Faculty of Medicine and Health Sciences, University of Barcelona, Barcelona, Spain: Adria Jorba-Garcia. Department of Oral surgery and implantology, Faculty of Medicine and Health Sciences, University of Barcelona, Barcelona, Spain: Octavi Camps-Font. Nano lab,Eye Transitional Research center,farabi Eye Hospital,Tehran University of Medical sciences,Tehran Iran: Ahad Khoshzaban. Department of Oral and Maxillofacial Surgery, “Diaz” Private Hospital, Padova, Italy: Aldo Zupi. Department of Oral and Maxillofacial Surgery, University Hospital LKH Salzburg, Salzburg, Austria: Alexander Johann Gaggl. Oral Surgery, Nantes University and University Hospital, Nantes, France: Anne-Gaëlle Chaux. Department of Oral and Maxillofacial Surgery, Federal Armed Forces Hospital, Koblenz, Germany; Oral and Maxilloafacial Surgery, University Medical Center; J. Gutenberg University, Mainz Germany: Anne-Kathrin Bär. Oral and Maxilloafacial Surgery, University Medical Center; J. Gutenberg University, Mainz Germany: Bilal Al-Nawas. Unit of Maxillofacial Surgery, Regional Hospital F. Miulli, University of Bari, Acquaviva delle Fonti, Bari, Italy: Antonio Mancini. Department of Oral Surgery, Gabriele d'Annunzio University of Chieti-Pescara, Italy: Antonio Scarano. Department of Oral and Maxillofacial Surgery, Ziekenhuis aan de stroom (ZAS), Antwerp, België: Casper Van den Borre. Private practice, Rome, Italy: Christian Alberti. Department of Oral and Maxillofacial Surgery and Centre for Reconstructive Surgery, University Hospital of the Private Medical University Paracelsus LKH, Salzburg, Austria: Christoph Steiner. Department of Oral and Maxillofacial Surgery and Centre for Reconstructive Surgery, University Hospital of the Private Medical University Paracelsus LKH, Salzburg, Austria: Gian Battista Bottini. Department of Oral and Maxillofacial Surgery, Head and Neck Universitary Institute, Nice Hospital, Nice, France: Cyril Debortoli. Integral Dentistry Department, Faculty of Dentistry, Universidad de La Frontera, Temuco, Chile: Eduardo Borie. Department of Dental Clinical Specialties. School of Dentistry. University Complutense of Madrid: Eduardo Montero. Department of Dentistry, Faculty of Medical Sciences, Albanian University, Tirana, Albania: Erda Qorri. Department of Oral and Maxillofacial and Periodontology, Ribeirão Preto School of Dentistry, University of São Paulo, Ribeirão Preto, Brazil: Erick Ricardo Silva. Department of Oral and Maxillofacial and Periodontology, Ribeirão Preto School of Dentistry, University of São Paulo, Ribeirão Preto, Brazil: Giuseppe Alexandre Romito. Faculty of Dental Sciences, Institute of Medical sciences. Banaras Hindu University. Varanasi,India: Farhan Durrani. Department of Oral and Maxillofacial Surgery, Royal Melbourne Hospital and Geelong University of Hospital: Felix Sim. Division of Oral Surgery, University Clinic of Dentistry, Medical University of Vienna, Vienna, Austria: Florian Beck. Department of Surgical Sciences, University of Turin, Turin, Italy: Giacomo Baima. Private practice Bad Oeynhausen, Germany: Jochen Tunkel. Department of Oral and Maxillofacial Surgery—Plastic Operations, University of Schleswig-Holstein, Campus Kiel: Henning Wieker. Department of Oral and Maxillofacial Surgery, Helios Hospitals, Kassel, Germany: Hendrik Terheyeden. Periodontology, Centre for Dental Education and Research, All India Institute of Medical Sciences, New Delhi, India: Kunaal Dhingra. Department of digital oral implantology and prosthodontics, College of Stomatology, Xi'an Jiaotong University, Xi'an, 710004, China: Liangzhi Du. Maxillo-Facial Surgery Unit, Department of Medicine, Surgery and Pharmacy, University of Sassari, Sassari, Italy: Luigi Angelo Vaira. Department for Oral and Maxillofacial Surgery, School of Dentistry, University of Dresen (TUD), Germany: Manfred Nilius. Dentistry Department, School of Health and Life Sciences, Pontifical Catholic University of Rio Grande do Sul (PUCRS), Porto Alegre, Brazil: Marcel Ferreira Kunrath. Department of Prosthodontics, Faculty of Dentistry, Cairo University, Giza, Egypt: Mariam Ahmed Roshdy. Department of Operative and Esthetic Dentistry, Faculty of Dentistry, University of Szeged, Szeged, Hungary: Mark Adam Antal. Private practice's owner and freelencer: Gregorio Redaelli. Department of of prosthodontics,faculty of dentistry, Mansoura University Mansoura,EGYPT: Mohamed Elgamal. Oral and Maxillofacial Department, Faculty of Dentistry, Menoufia University: Mohamed T. Khater. Department of Prosthodontics, Faculty of Dentistry, Menoufia National University, Menoufia, Egypt: Mohammed A. El-Sawy. Department of Oral Medicine, Periodontology and Oral Diagnosis,Faculty of Dentistry, AinShams University, Cairo, Egypt: Mohammed Samir. Department of Implant Dentistry, College of Stomatology, Xi'an Jiaotong University: Ningbo Zhao. CL Dentistry, Private Practice, Segni, Italy: Paolo Carosi. Department of Prosthodontics, New Horizon Dental College & Research Institute, Bilaspur, Chattisgarh, India: Gaurav Tripathi. Department of Periodontics and Oral Medicine, University of Michigan School of Dentistry, Ann Arbor, MI, USA: Hamoun Sabri. Department of Periodontics and Oral Medicine, University of Michigan School of Dentistry, Ann Arbor, MI, USA: Hom-Lay Wang.
