# Supplementary material for: Expected clinical and patient-centered outcomes associated with different rehabilitation strategies for severe maxillary atrophy: an international cross-sectional survey
Source: Front Oral Health. 2026 Sep 16;7:1873284. doi: 10.3389/froh.2026.1873284 (PMC13625680; doi:10.3389/froh.2026.1873284)
Supplement: Supplementary file 1 [file Supplementaryfile1.docx]

**Supplementary Material 1**

**Complete Survey Questionnaire**

Title of the survey: Maxillary Atrophy Class VI Cawood and Howell: How to Perform Implant-Prosthetic Rehabilitation?

This supplementary material reports the complete questionnaire used for the international cross-sectional survey. The questionnaire was administered through Google Forms and included an eligibility screening question, author/contact information fields, a standardized clinical case, general decision-making questions, and branching sections according to the therapeutic strategy selected by the respondent.

**Section 1. Eligibility Screening**

**1. How many complex rehabilitations did you complete in 2024?**

Response format: Single-choice

- <5
- 5–10
- >10

*Note: Respondents reporting fewer than five complex rehabilitations per year were directed to an exit section and excluded from the final analysis.*

**Section 2. Participation and Author Information**

**2. Do you agree to participate in this collaborative study?**

Response format: Single-choice

- Yes
- No

**3. Full name**

Response format: Free text

*Note: Format requested: first name, middle name/initial if applicable, last name.*

**4. Institutional affiliation**

Response format: Free text

*Note: Department/Unit, Faculty/Division, University/Hospital/Institution, City, Country.*

**5. Email address**

Response format: Free text

*Note: Professional/institutional email preferred; personal email accepted.*

**6. ORCID iD**

Response format: Free text

*Note: Optional but recommended.*

**Section 3. Standardized Clinical Case**

Respondents were presented with a standardized case of an 82-year-old female patient with severe maxillary atrophy requiring fixed implant-supported rehabilitation. The clinical information provided included sex, age, ASA status, relevant medical history, current medications, financial considerations, and the patient request for fixed implant-supported prosthetic rehabilitation.

A link to the anonymized CT/CBCT dataset was provided in the online questionnaire to allow respondents to evaluate the case using their own planning systems.

**7. Confirmation of access to the CT/CBCT scan**

Response format: Checkbox

- Ok

**Section 4. General Therapeutic Preference Questions**

**8. In this case, what is your preferred technique?**

Response format: Single-choice

- Bone grafting (autograft/xenograft) + traditional implants
- Alternative implantology (pterygoid/zygomatic/transnasal/tilted/subperiosteal/piezoimplant)
- Combination of both
- Other

**9. What factors most influence your choice?**

Response format: Multiple-choice

- Rehabilitation time
- 5-year implant survival rate
- Costs for the patient
- Surgical complexity
- Other

**10. In your experience, which technique provides the best aesthetic results?**

Response format: Single-choice

- Bone graft + traditional implants
- Alternative implantology (e.g., zygomatic/pterygoid)
- No difference
- I don't have sufficient data

**11. Which approach has, according to you, the highest 5-year survival rate in severe atrophies?**

Response format: Single-choice

- Bone grafts + traditional implants
- Alternative implants
- I don't have sufficient data

**12. Which technique, according to you, requires more long-term prosthetic revisions?**

Response format: Single-choice

- Bone grafts + traditional implants
- Alternative implants (e.g., All-on-4/zygomatic)
- No difference
- I don't have sufficient data

**13. The occlusal load distribution is more physiological with:**

Response format: Single-choice

- Traditional implants in axial position
- Alternative implants with proper balancing
- I don't notice clinical differences

**14. Choose your preferred rehabilitation technique in the provided case.**

Response format: Single-choice

- Alternative implant
- Bone graft and traditional implant
- Both
- Bone graft, traditional implant and alternative implant where necessary

*Note: This item determined the branching pathway for the subsequent strategy-specific questions.*

**Branch A. Hybrid/Combined Strategy**

**1. Could you please provide a brief description of your implant-prosthetic project?**

Response format: Free text

**2. Is your technique:**

Response format: Multiple-choice

- Safer (lower intraoperative risks)
- More predictable (better control of the outcome)
- Well tolerated by the patient (high morbidity)
- Not applicable in these cases
- Other

**3. Does your technique allow for immediate loading with a fixed prosthesis in complex cases?**

Response format: Single-choice

- Yes, always/usually
- Only in selected cases
- No, it requires a long time

**4. What is the expected 5-year survival rate for your technique in severe atrophy?**

Response format: Single-choice

- >95%
- 85–94%
- <85%

**5. Does your technique require complex/expensive prosthetics?**

Response format: Single-choice

- Yes, always
- No, standard
- Depends on the case

**6. Is your technique more prone to prosthetic complications (e.g., fractures, screw loosening)?**

Response format: Single-choice

- Yes, frequently
- No, it is stable
- Only in case of planning errors

**7. Considering the use of the same materials for each technique, is your chosen technique more expensive compared to other techniques?**

Response format: Single-choice

- Yes, significantly
- No, it costs the same as others
- Depends on the materials used

**8. The total treatment time, from surgery to functional loading, is:**

Response format: Single-choice

- <72 hours (immediate loading)
- 3–6 months
- 1 year

**9. In your experience, what is the patient satisfaction level 1 year after prosthetic loading?**

Response format: Single-choice

- Very high (>90% of patients satisfied)
- Moderate (70–90% satisfied)
- Low (<70% satisfied)
- Data not available

**10. The majority of post-treatment complaints involve:**

Response format: Multiple-choice

- Pain/prosthetic discomfort
- Aesthetic problems (e.g., unnatural gingival profile)
- Chewing/speech difficulties
- Surgical complications (e.g., infections)
- Other

**11. How much does the quality of life (Oral Health Impact) improve for patients after treatment?**

Response format: Single-choice

- Significant improvement
- Moderate improvement
- No noticeable change

**12. Do patients report difficulty with daily oral hygiene with your technique?**

Response format: Single-choice

- Yes, often (requires special tools)
- Only initially
- No, it is as simple as with natural teeth

**13. Are patients satisfied with the appearance of their smile (naturalness, lip profile)?**

Response format: Single-choice

- Yes, in most cases
- Only if the upper lip does not expose the gum
- No, they often require touch-ups

**14. Is the chewing function comparable to natural teeth?**

Response format: Single-choice

- Yes, completely
- Almost, with minor limitations
- No, considerable differences

**15. The post-operative discomfort lasts on average:**

Response format: Single-choice

- <1 week
- 1–4 weeks
- 1 month

**16. How many patients abandon treatment due to high morbidity?**

Response format: Single-choice

- <5%
- 5–15%
- >15%

**17. Is the annual maintenance more expensive compared to other techniques?**

Response format: Single-choice

- Yes, for frequent check-ups
- No, it is comparable
- Depends on the patient

**Branch B. Alternative Implant Strategies**

**1. Could you please provide a brief description of your implant-prosthetic project?**

Response format: Free text

**2. Is your technique:**

Response format: Multiple-choice

- Safer (lower intraoperative risks)
- More predictable (better control of the outcome)
- Well tolerated by the patient (high morbidity)
- Not applicable in these cases
- Other

**3. Does your technique allow for immediate loading with a fixed prosthesis in complex cases?**

Response format: Single-choice

- Yes, always/usually
- Only in selected cases
- No, it requires a long time

**4. What is the expected 5-year survival rate for your technique in severe atrophy?**

Response format: Single-choice

- >95%
- 85–94%
- <85%

**5. Does your technique require complex/expensive prosthetics?**

Response format: Single-choice

- Yes, always
- No, standard
- Depends on the case

**6. Is your technique more prone to prosthetic complications (e.g., fractures, screw loosening)?**

Response format: Single-choice

- Yes, frequently
- No, it is stable
- Only in case of planning errors

**7. Considering the use of the same materials for each technique, is your chosen technique more expensive compared to other techniques?**

Response format: Single-choice

- Yes, significantly
- No, it costs the same as others
- Depends on the materials used

**8. The total treatment time, from surgery to functional loading, is:**

Response format: Single-choice

- <72 hours (immediate loading)
- 3–6 months
- 1 year

**9. In your experience, what is the patient satisfaction level 1 year after prosthetic loading?**

Response format: Single-choice

- Very high (>90% of patients satisfied)
- Moderate (70–90% satisfied)
- Low (<70% satisfied)
- Data not available

**10. The majority of post-treatment complaints involve:**

Response format: Multiple-choice

- Pain/prosthetic discomfort
- Aesthetic problems (e.g., unnatural gingival profile)
- Chewing/speech difficulties
- Surgical complications (e.g., infections)
- Other

**11. How much does the quality of life (Oral Health Impact) improve for patients after treatment?**

Response format: Single-choice

- Significant improvement
- Moderate improvement
- No noticeable change

**12. Do patients report difficulty with daily oral hygiene with your technique?**

Response format: Single-choice

- Yes, often (requires special tools)
- Only initially
- No, it is as simple as with natural teeth

**13. Are patients satisfied with the appearance of their smile (naturalness, lip profile)?**

Response format: Single-choice

- Yes, in most cases
- Only if the upper lip does not expose the gum
- No, they often require touch-ups

**14. Is the chewing function comparable to natural teeth?**

Response format: Single-choice

- Yes, completely
- Almost, with minor limitations
- No, considerable differences

**15. The post-operative discomfort lasts on average:**

Response format: Single-choice

- <1 week
- 1–4 weeks
- 1 month

**16. How many patients abandon treatment due to high morbidity?**

Response format: Single-choice

- <5%
- 5–15%
- >15%

**17. Is the annual maintenance more expensive compared to other techniques?**

Response format: Single-choice

- Yes, for frequent check-ups
- No, it is comparable
- Depends on the patient

**Branch C. Bone Grafting and Traditional Implants**

**1. Could you please provide a brief description of your implant-prosthetic project?**

Response format: Free text

**2. Is your technique:**

Response format: Multiple-choice

- Safer (lower intraoperative risks)
- More predictable (better control of the outcome)
- Well tolerated by the patient (high morbidity)
- Not applicable in these cases
- Other

**3. Does your technique allow for immediate loading with a fixed prosthesis in complex cases?**

Response format: Single-choice

- Yes, always/usually
- Only in selected cases
- No, it requires a long time

**4. What is the expected 5-year survival rate for your technique in severe atrophy?**

Response format: Single-choice

- >95%
- 85–94%
- <85%

**5. Does your technique require post-operative surgical touch-ups?**

Response format: Single-choice

- Yes, frequently
- Rarely
- Almost never

**6. Is your technique more prone to prosthetic complications (e.g., fractures, screw loosening)?**

Response format: Single-choice

- Yes, frequently
- No, it is stable
- Only in case of planning errors

**7. Considering the use of the same materials for each technique, is your chosen technique more expensive compared to other techniques?**

Response format: Single-choice

- Yes, significantly
- No, it costs the same as others
- Depends on the materials used

**8. The total treatment time, from surgery to functional loading, is:**

Response format: Single-choice

- <72 hours (immediate loading)
- 3–6 months
- 1 year

**9. In your experience, what is the patient satisfaction level 1 year after prosthetic loading?**

Response format: Single-choice

- Very high (>90% of patients satisfied)
- Moderate (70–90% satisfied)
- Low (<70% satisfied)
- Data not available

**10. The majority of post-treatment complaints involve:**

Response format: Multiple-choice

- Pain/prosthetic discomfort
- Aesthetic problems (e.g., unnatural gingival profile)
- Chewing/speech difficulties
- Surgical complications (e.g., infections)
- Other

**11. How much does the quality of life (Oral Health Impact) improve for patients after treatment?**

Response format: Single-choice

- Significant improvement
- Moderate improvement
- No noticeable change

**12. Do patients report difficulty with daily oral hygiene with your technique?**

Response format: Single-choice

- Yes, often (requires special tools)
- Only initially
- No, it is as simple as with natural teeth

**13. Are patients satisfied with the appearance of their smile (naturalness, lip profile)?**

Response format: Single-choice

- Yes, in most cases
- Only if the upper lip does not expose the gum
- No, they often require touch-ups

**14. Is the chewing function comparable to natural teeth?**

Response format: Single-choice

- Yes, completely
- Almost, with minor limitations
- No, considerable differences

**15. The post-operative discomfort lasts on average:**

Response format: Single-choice

- <1 week
- 1–4 weeks
- 1 month

**16. How many patients abandon treatment due to high morbidity?**

Response format: Single-choice

- <5%
- 5–15%
- >15%

**17. Is the annual maintenance more expensive compared to other techniques?**

Response format: Single-choice

- Yes, for frequent check-ups
- No, it is comparable
- Depends on the patient

**Exit Section**

Respondents who completed the questionnaire or were not eligible according to the predefined experience threshold were directed to a final thank-you message.

**Figure 1**

**Figure 1. Standardized anonymized CBCT case used for treatment-planning assessment.**

Representative cone-beam computed tomography (CBCT) images of the standardized clinical case presented to all survey participants. The figure includes coronal sections at different anteroposterior levels, axial sections at different craniocaudal levels, a sagittal reconstruction, and a three-dimensional volume rendering. The images demonstrate severe maxillary atrophy consistent with Cawood and Howell Class VI and served as the radiological basis for therapeutic decision-making within the survey. All patient-identifiable information was removed prior to dissemination.

**Figure 2**

**Figure 2. Flowchart of participant selection and therapeutic preference allocation.**

A total of 178 questionnaires were submitted. After application of the predefined eligibility criterion requiring self-reported experience in at least five complex implant-prosthetic rehabilitations per year, 23 questionnaires were excluded. The final analysis included 155 eligible clinicians from 33 countries. Participants were allocated into three therapeutic preference groups according to the treatment strategy selected for the standardized CBCT case: alternative implant strategies (n = 83, 53.5%), regenerative approaches with bone grafting (n = 45, 29.0%), and hybrid/combined strategies (n = 27, 17.4%).
